# Factors Influencing Contraceptive Use Among Women With Serious Mental Illness Residing in Pennsylvania

**DOI:** 10.1111/psrh.70067

**Published:** 2026-05-31

**Authors:** Cara Nikolajski, Kelly Williams, Mary Hawk, Jessica G. Burke

**Affiliations:** ^1^ Department of Behavioral and Community Health Sciences School of Public Health, University of Pittsburgh Pittsburgh Pennsylvania USA; ^2^ Department of Infectious Diseases and Microbiology School of Public Health, University of Pittsburgh Pittsburgh Pennsylvania USA

## Abstract

**Introduction:**

Women living with a serious mental illness (SMI) can experience a myriad of challenges throughout their reproductive years including suboptimal contraceptive use and unintended pregnancy. Little is known about the contextual factors that influence the contraceptive use of women with SMI. To address this gap, we conducted qualitative interviews with women with SMI residing in Pennsylvania, to understand their pregnancy intentions, current contraceptive use, and factors that influence their contraceptive use behaviors and experiences.

**Methods:**

We conducted twenty‐eight semi‐structured in‐depth interviews with a convenience sample of women who were sexually active and between the ages of 18–45 with a diagnosis of bipolar disorder (*n* = 10), MDD (*n* = 12), and schizophrenia/schizoaffective disorder (*n* = 6). Two coders analyzed transcripts using Crabtree and Miller's editing approach to identify qualitative themes and subthemes.

**Results:**

Most women (89%) reported wanting to avoid pregnancy; however, their contraceptive use did not always align with these intentions. SMI symptoms, contraceptive knowledge and attitudes, substance use, reproductive coercion, intimate partner violence, and sexual assault/abuse influenced participants' contraceptive use. Among this sample, a larger proportion of women with schizophrenia/schizoaffective disorder experienced reproductive coercion compared to women with MDD or bipolar disorder.

**Discussion:**

Study findings underscore the critical need for contraceptive counseling that considers the complex and potentially unique challenges faced by women with SMI. Future research should focus on developing and evaluating integrated care models that effectively support their family planning and contraceptive needs.

## 1 Introduction

Serious mental illnesses (e.g., bipolar disorder, major depressive disorder (MDD), schizophrenia/schizoaffective disorder) are chronic and can result in functional impairment and limitations in major life activities [1]. Over 15 million adults in the United States live with a serious mental illness (SMI) [2], and over 3.5 million are women of reproductive age (defined here as 18–45 years of age) [3]. Women with SMI can experience a myriad of challenges throughout their reproductive years, such as suboptimal family planning counseling [4, 5, 6, 7], higher rates of negative pregnancy outcomes [8, 9], and an increased likelihood of child custody loss [10, 11, 12, 13]. In addition, unintended pregnancy, or a pregnancy that is mistimed, unexpected, or unwanted, is common among this population [14, 15], and can result in negative health and social consequences for mothers, children, and families [16].

Women can avoid unintended pregnancy by using effective contraceptive methods correctly and consistently [17, 18]; however, women with SMI often report low use or inconsistent contraceptive use [19, 20, 21, 22]. Existing studies show that, compared to the general population, women with SMI may be less likely to use any form of contraception [20, 22], more likely to rely on less effective methods such as condoms and hormonal pills [19, 22], and have a higher likelihood of becoming pregnant even after initiating contraceptive use [23].

Although a growing body of research has examined reasons for contraceptive use and nonuse among women with SMI, much of this work is dated or conducted outside of the United States, which may limit its applicability given advances in contraception options, evolving clinical practices, and differences in healthcare systems. Miller's 1998 study of women with schizophrenia/schizoaffective disorder found that participants were significantly more likely to admit “not getting around” to using contraception, consider it “too hard to take,” and experience delusional thinking about contraception compared to women without SMI [24]. Farr et al.'s [25] research of over 53,000 women who were at risk for pregnancy found that women with MDD commonly reported reasons such as cost, not thinking about the possibility of pregnancy, and pregnancy ambivalence as contributors to contraceptive nonuse.

Few studies provide information about contraceptive knowledge and attitudes of women with SMI. Those that do exist reveal that women with schizophrenia/schizoaffective disorder are less knowledgeable about contraceptive methods, including long acting reversible contraception (LARC), than women with other forms of SMI as well as those without SMI [24, 26]. Bursalioglu et al. [22] found no significant differences between contraceptive attitudes of participants with SMI and controls among a sample of Turkish women; however, no information was provided about what contraceptive methods were assessed and the specific questions that were asked to understand women's attitudes.

In addition, little is known about how reproductive coercion intersects with SMI to shape women's pregnancy and contraceptive experiences. Existing reproductive coercion research highlights important racial and ethnic disparities, socioeconomic contributors, and other factors associated with increased risk of experiencing reproductive coercion [27]; however, the role of mental health status has largely been overlooked. For example, Miller and colleagues found that African American women were twice as likely to experience pregnancy coercion and four times as likely to experience birth control sabotage compared to white women [28]. Similarly, Clarke et al.'s analysis of survey data from 641 women identified higher rates of reproductive coercion among women who were single, multiracial, and who experienced intimate partner violence (IPV) [29]. To date, however, there are no known qualitative studies that explore how reproductive coercion is experienced and interpreted by women with SMI.

Hall and Steinberg developed guidance on contraceptive counseling for women with SMI, offering important considerations to support shared decision‐making that reflects the unique reproductive needs of this population [30]. This guidance, and contraceptive counseling practices more broadly, can be further informed by additional qualitative research that examines how women with SMI navigate contraceptive use, decision making, and reproductive autonomy. Specifically, additional research is needed to understand the contextual factors, including biological factors like SMI symptoms; individual knowledge, attitudes, and behaviors; interpersonal relationships; and social‐structural determinants that influence the contraceptive use (or nonuse) of women with SMI. To address this existing knowledge gap, we conducted qualitative interviews with women residing in Pennsylvania who are of child‐bearing age with bipolar, MDD, and schizophrenia/schizoaffective disorders to understand their pregnancy intentions, current contraceptive use, and the factors that influence their contraceptive behaviors and experiences. In addition, we explored differences in contraceptive experiences across SMI diagnosis categories.

## 2 Methods

### 2.1 Data Collection

We used several strategies to identify and enroll eligible participants, including displaying study flyers in outpatient mental health treatment facilities, conducting brief study overview presentations during intensive outpatient treatment program sessions, and soliciting provider referrals. Eligible women had a self‐disclosed diagnosis of bipolar disorder, MDD, or schizophrenia/schizoaffective disorder; were between the ages of 18–45; spoke fluent English; and had sexual intercourse within the previous year. Although we did not verify patient diagnoses through medical record review, all participants with schizophrenia/schizoaffective disorder had their diagnosis confirmed by providers at the time of referral. All other participants were recruited from intensive outpatient treatment groups organized by diagnosis (e.g., groups specifically for individuals with bipolar disorder or MDD). We conducted and recorded all interviews in a private room at the outpatient location where participants were receiving mental health treatment services. Participants completed a brief questionnaire at the end of their interview which captured demographic information, SMI diagnosis, and pregnancy history. We classified participant SMI diagnosis into mutually exclusive diagnostic categories based on clinical complexity (e.g., difficult to treat, typical symptom severity) for our three conditions of interest, with schizophrenia/schizoaffective disorder viewed as the highest level of complexity and MDD as lowest. We chose this hierarchical categorization based on psychiatrist feedback and review of the literature [31, 32]. We categorized participants experiencing co‐occurring mental health conditions into a single diagnostic category according to this hierarchy to allow for mutually exclusive analytic comparisons that reflect the most clinically complex diagnosis rather than the absence of co‐occurring conditions.

We developed the interview guide based on previous research conducted by Borrero et al. [33] which explored contraceptive use and family planning experiences of low‐income women. We asked women about their current pregnancy intentions by asking, “What would it be like if you got pregnant right now?” and using follow‐up probing questions. We also asked about contraceptive use and included follow‐up questions to understand women's reasons for using or not using contraception. Additionally, we asked women about their attitudes toward several contraceptive methods, experiences with reproductive coercion (e.g., birth control sabotage or partner control of pregnancy outcomes), sexual relationship experiences, and pregnancy planning. To decrease the risk of discomfort for participants, we did not ask specific questions regarding substance use and sexual abuse/assault; however, these topics often came up organically through conversation. Interviews lasted an average of 56 min. Participants completed a brief demographic questionnaire at the end of the interview and received a $25 gift card for their time. The study protocol was reviewed and approved by the authors' institutional Human Research Protection Office (PRO16070518).

### 2.2 Data Analysis

We transcribed all recordings verbatim; however, identifiable information was removed prior to analysis to ensure confidentiality. We used Crabtree and Miller's editing approach as our analytic framework. In brief, this approach involves identifying meaningful segments of transcript text, iteratively developing and refining codes, and ultimately organizing related codes into overarching themes and subthemes to capture central ideas or experiences emerging from the data [34]. We developed most codes inductively and without a predefined framework; however, we based some codes on Borrero et al.'s [33] pregnancy intention categorizations, including: wanting to avoid pregnancy, desiring pregnancy, ambivalence about pregnancy desires, and lacking intention. We selected this framework because it moves beyond the traditional and overly simplified categorization of intended versus unintended pregnancy, enabling us to capture the complexity of reproductive intentions, including ambivalence and lack of intention. To assess current contraceptive use, we divided contraceptive methods into three primary categories that are commonly used to describe their effectiveness in preventing pregnancy, including: low effectiveness (e.g., condoms, withdrawal), moderate effectiveness (e.g., hormonal pill, patch, vaginal ring, injection), and high effectiveness (e.g., intrauterine device (IUD), contraceptive implant). We used this categorization because it remains a widely recognized structure that provides a clear and practical way to describe contraceptive methods by their relative ability to prevent pregnancy [35].

During the analytic process, we organized transcript text into segments comprised primarily of responses to specific interview questions. We then applied codes to each segment of text. We used qualitative analysis software (Dedoose) [36] to support coding and organization of study data. To promote intercoder reliability, two independent analysts coded a portion of the study narratives. Following this process, the analysts met to discuss their coding decisions and discrepancies. The remaining narratives were coded independently, and the two coders then reconvened to discuss coding decisions and talk through any disagreements until a final code(s) was applied to each portion of the text being analyzed. Like codes were categorized into the themes described in the results below. We used our demographic questionnaire to link participant characteristics with corresponding qualitative transcripts to explore themes across patient subgroups (e.g., SMI diagnosis).

## 3 Results

We conducted twenty‐eight interviews with women with bipolar disorder (*n* = 10), MDD (*n* = 12), and schizophrenia/schizoaffective disorder (*n* = 6) from December 2016 to May 2017. Participants had an average age of 28.4, 70% were white, all had either a high school diploma or GED, and 65% took at least some college classes. Over 60% of participants had an income of less than $30,000/year and 68% were covered by public insurance. The majority (82%) were single, 14% were married, 4% were divorced, and 32% were currently living with a male sexual partner. Slightly over half of the study sample (54%) were never pregnant, and of the remaining 46% who conceived one or multiple times, 25% continued a pregnancy to term. Table 1 provides an overview of participant characteristics. The themes discussed below provide information about participants' current pregnancy intentions and contraceptive use, and the factors that influenced their use or nonuse of contraception.

**TABLE 1:** Participant characteristics.

Participant characteristics	*N* (%)
Mean age (range)	28.5 (18–45)
Race	
White	19 (68)
Black/African American	7 (25)
More than one race	2 (7)
Hispanic/Latino	
Yes	1 (4)
No	27 (96)
Education level	
High school	3 (11)
Associates degree	1 (4)
Some college	18 (64)
College and/or graduate degree	6 (21)
Household income	
$0–$9999	10 (36)
$10,000–$29,999	7 (25)
$30,000–$49,999	2 (7)
$50,000–$69,999	1 (4)
$100,000 or more	6 (21)
Missing	2 (7)
Insurance type	
Public/Medicaid	19 (68)
Private/Commercial	9 (32)
Primary SMI diagnosis	
Major depressive disorder	12 (43)
Bipolar disorder	10 (36)
Schizophrenia/schizoaffective disorder	6 (21)
Other mental health condition	
Yes	10 (36)
No	18 (64)
Years since SMI diagnosis	
< 1 year	6 (21)
1–10 years	12 (43)
> 10 years	9 (32)
Missing	1 (4)
Marital status	
Single	22 (79)
Married	4 (14)
Divorced	2 (7)
Living with sexual partner	
Yes	10 (36)
No	18 (64)
# of children	
0 children	21 (75)
1–2 children	5 (18)
3 or more children	2 (7)
# of pregnancies	
0 pregnancies	15 (54)
1–2 pregnancies	6 (21)
3 or more pregnancies	7 (25)

### 3.1 Current Pregnancy Intentions and Rationale

All but three women indicated that they wanted to avoid pregnancy. Women wanted to avoid pregnancy for several reasons, including (1) other priorities: “I think it would be hard to go to school and have a child at the same time.” (age 20, MDD); (2) financial instability: “I'd want to be good financially and be able to provide my child with, not even luxuries, but basic needs.” (age 20, bipolar disorder); and (3) not having a spouse: “It obviously has to be after marriage. I wouldn't want to start a family without a husband.” (age 18, bipolar disorder).

Women also mentioned reasons for wanting to avoid pregnancy that were directly related to their SMI symptoms and experiences. Some felt as though their SMI symptoms may not allow them to effectively care for a child, as one woman with MDD and self‐disclosed obsessive‐compulsive disorder (OCD) stated:

I don't think I can mentally handle having a child, because with the OCD, a lot of the thoughts are about children sometimes. Like harming them…I just don't think I can go through that again. It's like, I have habituated to my son…so I don't have to worry about that at all. But if I had another kid or adopted a baby, all the thoughts would come back, and it would be like opening a fresh wound. And then when I get depressed and I don't want to do anything, I can't just leave a young child to fend for themselves, so I'd have to force myself. I just don't think I would be good for it mentally. (age 40, MDD).

Other participants were hesitant about having or adamantly did not want to have children because they feared their SMI was hereditary. As two women shared: “I don't want to have the potential of even passing on bipolar.” (age 30, bipolar disorder) and “I don't want kids. I don't want to pass on what I have to another living being. I would never want somebody to live the life that I'm living.*”* (age 36, MDD) Other women wanted to be sure that their SMI symptoms were under control prior to conceiving: “I'm not ready for that…I want to be stable. I want to have been stable for a while and I want to have the blessings of my doctors. I also want to be on meds that are known not to mess up a kid.” (age 26, bipolar disorder).

Of the three women who did not indicate that they were trying to avoid pregnancy, one desired pregnancy, one was ambivalent, and one was lacking any pregnancy intention. The participant who was certain that she wanted to become pregnant stopped using contraception because she was over the age of 35 and a smoker, both factors that can contraindicate the use of some hormonal methods of birth control. After considering her current pregnancy intentions, she explained: “I feel like I'm at a point in my life where I can give a lot to a child if I were to become pregnant. I want a child. That is my angle, to bring life into this world. I would be so happy…I got a lot of wisdom to impart.” (age 38, schizophrenia/schizoaffective disorder) Pregnancy ambivalence was only felt by one woman who had been trying for almost a year to become pregnant again with mixed emotions: “I was thinking of going back on birth control but then whenever it gets to the point of actually doing it, I wimp out. ‘Cause I want a kid and then the other part—and there's times that I don't want a kid. Like, right now, I want a kid, but then I know I have to wait’ ‘cause I have a lot of stuff going on. All that stress can hurt the fetus.’” (age 19, bipolar disorder) One woman did not desire to become pregnant or avoid pregnancy, nor was she ambivalent about having a child. She realized that it was a possibility, but felt that pregnancy was ultimately outside of her control and, thus, lacked any sort of pregnancy intention:

I don't think about it. I just know that being as though me and [partner's name] are having unprotected sex, I know that, you know, it's a possibility that one day it could possibly happen…I wouldn't be mad, I would just be like, ‘Wow, I'm about to have a baby again after all this time. But I really don't think God has that for me though. I really don't. It's been 10 years and we've never ever got pregnant. (age 37, MDD).

### 3.2 Current Contraceptive Use

Twenty women (71.4%) were using some form of reversible contraception. Of these women, 10 (50%) reported using methods with lower effectiveness rates (e.g., condoms, withdrawal), seven (35%) relied on moderately effective methods (e.g., hormonal pill, patch, vaginal ring, injection), and three (15%) were using LARC (e.g., IUD, contraceptive implant). Two women had a hysterectomy, one woman's partner had a vasectomy, and two were not using contraception to prevent pregnancy because their current relationships did not pose a pregnancy risk (one participant was a transgender female and another was in a relationship with a transgender male—both self‐disclosed after enrollment). Table 2 provides an overview of contraceptive use for each SMI diagnosis category. Emergency contraception, or the “morning after pill”, was not assessed as a current method and is therefore not included in Table 2, despite reports of periodic use by some participants.

**TABLE 2:** Current contraceptive use by SMI diagnosis.

SMI diagnosis➔ ↓Contraceptive method	Bipolar disorder *N* (%)	Major depressive disorder *N* (%)	Schizophrenia/Schizoaffective disorder *N* (%)	Total *N* (%)
None	2 (20.0%)	1 (8.3%)	0 (0.0%)	3 (10.7%)
Low effectiveness	5 (50.0%)	2 (16.7%)	3 (50.0%)	10 (35.7%)
Moderate effectiveness	0 (0.0%)	5 (41.7%)	2 (33.3%)	7 (25.0%)
High effectiveness	2 (20.0%)	1 (8.3%)	0 (0.0%)	3 (10.7%)
Other	1 (10.0%)	3 (25.0%)	1 (16.7%)	5 (17.9%)

Women's pregnancy intentions did not always align with their contraceptive use. Seven of the 10 women relying on condoms and withdrawal wanted to avoid pregnancy; however, they were not using these methods consistently, which increased their pregnancy risk. Three women were not using any form of contraception. Participants identified several factors beyond pregnancy intention that influenced their contraceptive use. These factors were categorized into the themes described below:

### 3.3 Determinants of Contraceptive Use/Nonuse

SMI symptoms, which influenced sexual behaviors and contraceptive choice, contraceptive knowledge and attitudes, substance use, reproductive coercion, IPV, and sexual assault/abuse, affected participants' contraceptive use.

#### 3.3.1 SMI Symptoms

Women with bipolar disorder and MDD reported that their SMI symptoms influenced their contraceptive behaviors. Many women with bipolar disorder cited experiences of mania as a common reason for not using contraception. Some women related experiences of extreme emotional highs with condom nonuse:

I think if I would be more manic then I would be more likely not to use a condom or just, you know, just not think that much about it…One of the symptoms of being a little more manic than normal would be taking dangerous sexual risks…I think your judgement is a little bit impaired when you're manic. (age 20, bipolar disorder).

Another woman described the impact of her fluctuating emotions and bipolar symptoms on sexual experiences and risk taking: “I think when I'm feeling manic, I'm more likely to not use one [a condom]. When I'm depressed, I just don't have sex.” (age 38, bipolar disorder) This participant recalled having at least 12 past pregnancies, none of which resulted in a live birth.

Women's choice of contraception was influenced by the perceived or suspected impact of contraception on SMI symptoms, especially among women with MDD. One participant was considering the contraceptive implant, but chose a less effective method after contemplating its potential effect on her symptoms:

I was originally thinking of getting the implant, the arm one. But then I talked to my doctor and, like, because it's an implant, it's a little harder to take out. And we don't know if it was going to impact my depression or not. So, the choice of the NuvaRing [vaginal ring] was [because] it's localized. So, it's less likely to impact my depression. (age 20, MDD).

Another participant stopped using contraception because she thought her current birth control pills were increasing her depression symptoms: “When I first got diagnosed, I was like, ‘What if it's the pill is causing me to be depressed?’ So, I was like, you know what, let me stop taking these pills…But it didn't change my depression…It's obviously not the pills.” She had since started using birth control pills again, but periodically *“takes breaks”* and relies only on condoms for several months due to a fear that “If I keep taking them [birth control pills] all of my life, then I won't be able to have children or something.” (age 20, MDD).

### 3.4 Contraceptive Knowledge & Attitudes

Most participants stated that they had at least some knowledge about the various methods of contraception available to them, including condoms, withdrawal, hormonal pill, patch, vaginal ring, and injection, LARC, and emergency methods including the morning after pill. However, two women with schizophrenia/schizoaffective disorder stated that they had very little knowledge about contraceptive methods when they first became sexually active and that this directly resulted in an unintended pregnancy. One woman feared that she would be permanently hospitalized because of her diagnosis and sought a partner to have sex with so she could “experience [sex] in case I ended up in the hospital again.” She continued to describe her first sexual experience: “Well, I did have a condom and I was trying to put it on…I didn't really know how to use them, but he tried putting it on and then we tried doing it but then it didn't work so I tried again, but it still didn't work so he got fed up with it and just took it off.” (age 32, schizophrenia/schizoaffective disorder) Similarly, a participant with schizophrenia/schizoaffective disorder explained how little she knew about pregnancy prevention methods prior to her first pregnancy:

I had no idea about anything. So, when it came to condoms, I had no idea about that…I had no idea that sex got you pregnant…I mean, if I had known that sex without condoms could lead to a baby, I would have made different choices…It was actually after I got pregnant that my ex‐husband started becoming abusive. So, after I had the baby, I started researching and gaining more knowledge so that I could make sure that I didn't have another baby. (age 32, schizophrenia/schizoaffective disorder).

IUDs and contraceptive implants, both forms of LARC, were the least commonly known contraceptive methods among the study sample. Three women had no knowledge about IUDs and nine women stated that they were unaware that a contraceptive implant was an available method. There was also variation in contraceptive knowledge by diagnosis category. Awareness of the contraceptive implant was lowest among participants with schizophrenia/schizoaffective disorder (*n* = 3, 50%). Women with bipolar disorder (*n* = 3, 30%) and women with MDD (*n* = 3, 35%) were also unfamiliar with it. Sources of information about LARC varied, with some participants mentioning learning about it from their doctor or the internet. Friends and family served as a primary source of information for participants who expressed apprehension about LARC use.

Despite the effectiveness of LARC in preventing pregnancy, participants mentioned several concerns related to LARC use. Primary reasons women chose not to have an IUD or implant inserted included fear of bodily harm. Several women echoed the following sentiments: “It scares me because I'm afraid that it will slip and puncture something. That's why I never went on it, but I had a doctor suggest it to me. I was like, no, because I don't want to take that risk.” Another woman questioned whether the IUD could affect her ability to become pregnant in the future: “Let's say it was to break or if it was to rip any lining or cause damage to the uterus. Then eventually you want to get pregnant, but you can't because that has caused damage to your uterus and then you're screwed. It scares me.” (age 32, schizophrenia/schizoaffective disorder).

Several women described their perceptions of the intrusive nature of IUDs and implants and that they did not want a foreign object inside of their uterus. One woman described her feelings about the IUD:

I don't like things being put up in my vagina because it's painful. I believe it's not part of something that should be, like, inside of you if it doesn't belong there…Condoms are the least intrusive, and the intrauterine devices are, for me, too intrusive. The thought of having to stick something up there and pull it out every once in a while. You can remove it after seven years. Like, seven years with…a foreign object in your body just didn't strike me as very practical or very safe…Maybe you can contract a disease, maybe the chemicals or whatever they use can affect your body or hormones…I hear all these stories. (age 32, schizophrenia/schizoaffective disorder).

This participant's attitudes about IUDs and her belief that, once inserted, a woman had to continue to rely on the IUD for the full duration of its effective lifespan contributed to her not considering a LARC method. Given this, she used only condoms (albeit inconsistently) for pregnancy protection.

Three women with bipolar disorder and two with schizophrenia/schizoaffective disorder (none with MDD) found the idea of an “implant” undesirable and, therefore, did not consider using this LARC method. Some participants made general statements like: “I don't think I would want that just because it's an arm implant. I don't think I would want an implant of anything.” One woman with bipolar disorder feared the implant's hormonal elements and likened it to science fiction:

I don't like the word “implant”. I don't want anything implanted. The word bothers me—implant—and then also the hormones in there, the hormones that are in it…And then it seems like such a major thing to do with your body…It's like sci‐fy‐y, like a robot and I don't want anything to do with that.

A woman with schizophrenia/schizoaffective disorder directly linked her thoughts about the contraceptive implant to her SMI symptoms: “It has to do with my delusions. Um, implants being, like, trackable by the government and that goes way deep. Implants, anything underneath the skin…I wouldn't do.” (age 32, schizophrenia/schizoaffective disorder).

Of all participants, only three women possessed generally positive attitudes toward IUDs and seven felt similarly about the contraceptive implant. Primary reasons women supported the use of LARC included increased effectiveness: “I think it would be more effective because not everybody remembers to take the pills.” (age 43, MDD) and convenience/duration of LARC: “I was interested in that because for five years, you wouldn't have to worry about birth control. ‘Cause it was a small incision in your arm and I was getting my Depo shots in my arm anyway. So, what would be a little sliver of something for five years?” (age 38, schizophrenia/schizoaffective disorder).

### 3.5 Substance Use

Six women associated substance use with their sexual and contraceptive behaviors and experiences, including contraceptive nonuse and unintended pregnancy. One participant stated: “I've had unprotected sex in the past. I think a lot of that has to do with the alcoholism and drug use.” (age 29, MDD) When asked why she used condoms with some sexual partners and not others, another participant stated: “It can depend on the influences, are there drugs or alcohol involved?” (age 20, bipolar disorder) A third woman shared her experiences with substance use that contributed to her unintended pregnancy:

I was doing drugs at the time. Um, marijuana, I was drinking, underage drinking, going to parties, having sex with random guys and, like, it started affecting me. I was getting sick, I wasn't eating right. So, when I went to the doctor, they're like, ‘You're pregnant,’ and I was like, ‘So, what do I do?’ (age 19, bipolar disorder).

Substance use led some participants to engage in sexual risk‐taking behaviors, making them vulnerable to unintended pregnancy.

### 3.6 Sexual Violence and Abuse

Although we did not specifically ask about sexual violence and abuse, several participants disclosed these experiences. Overall, 18 of 28 study participants (64%) disclosed experiencing some form of IPV including reproductive coercion, childhood sexual abuse, or sexual assault/rape. Some women shared information about their experiences with multiple forms of abuse in their lifetime. Approximately half of the women in the study sample with MDD and bipolar disorder and all participants with schizophrenia/schizoaffective disorder (*n* = 6) experienced physical and/or sexual violence.

#### 3.6.1 IPV and Reproductive Coercion

Eight women disclosed being physically or emotionally abused by an intimate partner and ten described experiences with reproductive coercion, specifically contraceptive sabotage (e.g., condom refusal, flushing birth control pills) and pregnancy pressure (e.g., pressuring a woman to become pregnant when she does not want to). Several women described experiences with condom refusal, including: “I had met some guy in a bar…and I had brought a condom. And he was like, ‘No, I am not going to use this.’” (age 40, MDD), and “When I got pregnant [it was because] he refused to use a condom. He just said, ‘Oh don't worry about it, don't use it.’ And he threw it to the ground.” (age 32, schizophrenia/schizoaffective disorder) Experiences with condom refusal ranged from male partners preferring the feeling of sexual intercourse without a condom to men who were actively promoting pregnancy.

Six women experienced pressure from sexual partners to become pregnant with one woman obtaining a protection from abuse order because of her partner's pregnancy insistence and anger: “He said, ‘Oh I want kids.’ And part of that anger, I think, is because I kept saying no to him about kids…He wouldn't accept that. He was getting mad.” (age 29, schizophrenia/schizoaffective disorder) Of the 10 women who experienced any form of reproductive coercion, three became pregnant because of their partner's pregnancy pressure or condom refusal. One woman with schizophrenia/schizoaffective disorder became pregnant at age 16 by her 21‐year‐old partner: “He did it on purpose…Because of his level of love or care for me and wanting to start a family and be with me…He actually proposed or wanted to propose and become engaged and get married and start a family.” She continued to explain that her partner's coercive behaviors did not result in one, but three pregnancies: “He did not want me to be on it [birth control] because his goal was to impregnate me. And that's when I got pregnant twice the following year at the age of 17.*”* (age 25, schizophrenia/schizoaffective disorder).

Another participant chose a specific method of contraception to avoid becoming pregnant by her former husband:

My ex‐husband was abusive, and he wanted me to get pregnant again. And so I ran to the clinic one day and I knew he was coming right after me and I just ran in and told them my husband's abusive and I need something right now to make sure I don't get pregnant and they gave me the Depo shot. Because, it was like, he would never know. He would find the pills, you know, that's why I ended up getting it [hormonal injection]. (age 32, schizophrenia/schizoaffective disorder).

She chose to use a hidden method of contraception to remain protected from pregnancy as she determined a way to leave her abusive relationship.

All six women with schizophrenia/schizoaffective disorder were among the ten women who disclosed personal experiences with pregnancy pressure and/or contraceptive sabotage. Of these six women, five were women of color, and three experienced an unintended pregnancy resulting from reproductive coercion. No participants with bipolar disorder or MDD attributed an unintended pregnancy to coercive behaviors by a sexual partner.

### 3.7 Childhood Sexual Abuse and Sexual Assault

Study participants commonly described experiences of childhood sexual abuse and sexual assault/rape. Four women (two with bipolar disorder, one with MDD, and one with schizophrenia/schizoaffective disorder) described experiences of sexual abuse during their childhood or teenage years. One woman described sexual experiences forced by her brother: “I was raped by my brother. Um, I was 15 when he raped me…And when I got pregnant with his kid, I told my mom…She didn't want to believe me and then whenever I went and got a DNA test done, she kicked me out and not him.” (age 19, bipolar disorder) Another participant was unable to continue using the vaginal ring for contraception because of her childhood sexual abuse experiences: “I did try the NuvaRing for one month and I didn't like it cause…like, I have PTSD from early age trauma…The NuvaRing is triggering.” (age 38, bipolar disorder).

Five additional participants described experiences of sexual assault and rape with one woman obtaining emergency contraception to prevent pregnancy after her sexual assault: “This happened last October, and it was a sexual assault. I didn't want to go to the hospital or anything right away and I didn't want to have to deal with any consequences, so I used that [emergency contraception] and that was almost an immediate thought that I needed to get that.” (age 21, MDD). Another participant who was repeatedly raped by her husband stated that she did not consider that she was being raped until she talked about these experiences with her therapist, who confirmed that her husband was, in fact, sexually abusing her.

These unwanted and often violent sexual experiences increased participants' risk for unintended pregnancy and sometimes influenced the contraceptive methods they used or discontinued.

## 4 Discussion

Most participants in this study expressed a desire to avoid pregnancy while navigating competing life priorities, including educational goals, financial stability, finding a partner, and management of SMI symptoms. Existing literature supports these and other reasons for delayed childbearing [37, 38, 39]. While LARC is highly effective at preventing unintended pregnancy [35], participants described concerns specific to the potential bodily harm and their lived experiences with SMI that contributed to negative attitudes toward these methods. Some participants expressed worries about possible uterine damage or the effects of IUD‐associated hormones on future fertility. Others raised concerns that the hormonal effects of an implant or IUD might exacerbate psychiatric symptoms or undermine their sense of bodily autonomy and control. Together, these concerns illustrate that LARC effectiveness alone was insufficient to offset the perceived risks.

Consistent with prior literature demonstrating that contraceptive decision‐making is influenced by factors beyond efficacy [40, 41], our findings underscore the importance of a patient‐centered approach that prioritizes individuals' values, preferences, and health‐related concerns. For people with SMI, respectful contraceptive counseling should acknowledge potential apprehension regarding symptom triggers and emphasize shared decision‐making rather than promoting any specific method based solely on pregnancy risk.

Over a third of participants (36%) in this study had a sexual partner who tried to pressure them to become pregnant or in some way refused to use or sabotaged contraception to promote pregnancy. Twenty‐five percent of these women experienced pregnancy pressure and 21% experienced birth control sabotage, with some women experiencing both. Comparatively, Miller's 2010 study of a sample of women ages 16–29 from the general population receiving services in California family planning clinics found that 19% and 15% of study participants experienced pregnancy pressure and birth control sabotage, respectively [28]. Although our study sample is small, our findings reveal that reproductive coercion may be a common experience for women with SMI. Further, all six participants with schizophrenia/schizoaffective disorder described personal experiences with reproductive coercion. Although these findings suggest that women with schizophrenia/schizoaffective disorder may be particularly vulnerable to these experiences, five of the six participants with these disorders were women of color, a group that has also been shown to be at increased risk for reproductive coercion [28, 42]. Taken together, these findings underscore the need for further research to examine how mental health status, race and ethnicity, and broader structural conditions may interact to shape women's reproductive experiences and contribute to reproductive health inequities.

Even though participants were not specifically asked about their experiences with IPV and sexual abuse, several women voluntarily disclosed these experiences. In addition to the lasting trauma that often results from sexual violence, these experiences contributed to an increased risk of, or a personal experience with, unintended pregnancy among study participants. This corresponds closely with existing literature which shows that women who experience abuse also experience increased rates of negative reproductive outcomes including unintended pregnancy and delayed prenatal care [43, 44, 45]. Several review articles describing experiences of IPV, domestic violence, and sexual abuse among women with SMI reveal alarming rates ranging from 21% to 76% [46, 47, 48]. Screening women with SMI for IPV and sexual abuse is critically important to determine whether they may be at increased risk for adverse reproductive experiences. Mental health treatment settings may play an important role in IPV assessment; however, further investigation is needed to determine how to train mental health clinicians to effectively detect IPV in a manner that minimizes patient discomfort or harm when disclosing this sensitive information.

This study highlights important directions for research and clinical practice. There are important opportunities to develop and evaluate care models that integrate mental health and reproductive health services in ways that reflect the needs and preferences of women with SMI. Findings suggest that contraceptive counseling should account for the influence of mental health symptoms, contraceptive knowledge, substance use, and experiences of abuse, trauma, and reproductive coercion on women's contraceptive experiences. Greater integration of contraception discussions within mental health treatment settings, along with clinician training in shared decision‐making and trauma‐informed care, may support counseling approaches that respect reproductive autonomy while acknowledging safety concerns and contextual barriers.

Several limitations should be considered, as they may affect the interpretation of study findings. First, the small sample size within each diagnostic category and the timing of data collection (2016/2017) may have limited our ability to achieve thematic saturation across categories and may not reflect recent changes in contraceptive care or counseling practices for women with SMI. Second, as described in the Methods, diagnostic categories were defined hierarchically for analytic purposes. Future research should conduct an in‐depth exploration of the contraceptive use experiences of women with co‐occurring mental health conditions to better capture the complexity of these experiences. Third, while our data show that women with schizophrenia/schizoaffective disorder may have less knowledge about LARC methods and may be more vulnerable to reproductive coercion, the study sample only included six women with these diagnoses. Additional interviews with participants with schizophrenia/schizoaffective disorders may have resulted in different conclusions. Fourth, other factors such as race/ethnicity, education, poverty status, and structural disadvantage may shape women's contraceptive use experiences alongside or apart from SMI diagnosis; however, the sample was too small to examine these intersecting influences, underscoring the need for further research into how these social‐structural factors relate to the contraceptive use experiences of women with SMI. Fifth, we collected participant characteristics, including SMI diagnosis, via self‐report, which may limit diagnostic precision.

Study findings support existing research indicating that women cannot always control their contraceptive use/nonuse and that pregnancy intentions may not align with contraceptive use. Symptoms of SMI, along with contraceptive knowledge and substance use, can impact women's ability to effectively use contraception. Additionally, reproductive coercion, sexual abuse, and IPV are significant issues among this population and may increase their risk of experiencing unintended pregnancy. All women deserve the right to informed and supported reproductive decision‐making that is free from discrimination and harm. This study underscores the critical need for contraceptive counseling that considers the complex and, at times, unique challenges faced by women with SMI, empowering them with the freedom of choice related to their contraceptive needs and desires.

## Conflicts of Interest

The authors declare no conflicts of interest.

## Data Availability

The data that support the findings of this study are available from the corresponding author upon reasonable request.
